# Emotion processing in youths with conduct problems: an fMRI meta-analysis

**DOI:** 10.1038/s41398-023-02363-z

**Published:** 2023-03-30

**Authors:** Kathryn Berluti, Montana L. Ploe, Abigail A. Marsh

**Affiliations:** 1 Department of Psychology, Georgetown University, Washington, DC USA; 2 Department of Psychology, Washington State University, Pullman, DC USA

**Keywords:** Diagnostic markers, Neuroscience

## Abstract

Functional magnetic resonance imaging (fMRI) studies consistently indicate differences in emotion processing in youth with conduct problems. However, no prior meta-analysis has investigated emotion-specific responses associated with conduct problems. This meta-analysis aimed to generate an up-to-date assessment of socio-affective neural responding among youths with conduct problems. A systematic literature search was conducted in youths (ages 10–21) with conduct problems. Task-specific seed-based d mapping analyses examined responses to threatening images, fearful and angry facial expressions, and empathic pain stimuli from 23 fMRI studies, which included 606 youths with conduct problems and 459 comparison youths. Whole-brain analyses revealed youths with conduct problems relative to typically developing youths, when viewing angry facial expressions, had reduced activity in left supplementary motor area and superior frontal gyrus. Additional region of interest analyses of responses to negative images and fearful facial expressions showed reduced activation in right amygdala across youths with conduct problems. Youths with callous-unemotional traits also exhibited reduced activation in left fusiform gyrus, superior parietal gyrus, and middle temporal gyrus when viewing fearful facial expressions. Consistent with the behavioral profile of conduct problems, these findings suggest the most consistent dysfunction is found in regions associated with empathic responding and social learning, including the amygdala and temporal cortex. Youth with callous-unemotional traits also show reduced activation in the fusiform gyrus, consistent with reduced attention or facial processing. These findings highlight the potential role of empathic responding, social learning, and facial processing along with the associated brain regions as potential targets for interventions.

## Introduction

Conduct problems, including aggression, non-violent delinquency, and other antisocial behaviors all increase rapidly during late childhood, spiking in adolescence and young adulthood [1]. However, youths who display particular patterns of activation when processing socio-affective information have a greater likelihood of exhibiting conduct problems across development [2–5]. Identifying neurodevelopmental features of the highest-risk youths is essential for developing targeted screening and intervention tools. To our knowledge, this is the first meta-analysis of youth with conduct problems to investigate patterns of emotion-specific neural responding [6–10].

Many youths with conduct problems receive diagnoses of conduct disorder (CD) or oppositional defiant disorder (ODD) [11]; either of these diagnoses before the age of 15, relative to all other childhood diagnoses, place youth at the highest risk of adult clinical diagnoses such as substance use disorder and antisocial personality disorder [12]. Other high-risk youths receive diagnoses of a disruptive behavior disorder not otherwise specified (DBD NOS) or exhibit subthreshold conduct problems that nonetheless are associated with increased delinquency, substance use, and aggression [13]. A subset of youths with behavior problems and callous-unemotional traits or limited prosocial emotions (LPE), characterized by reduced guilt and empathy, and a bold, fearless temperament, exhibit more homogenous patterns of neural risk factors [11]. These traits are highly correlated with externalizing behaviors such as aggression, bullying, and delinquency and have poorer treatment outcomes [14, 15].

Neuroimaging research has identified aberrant brain responses in youth with conduct problems with and without callous-unemotional traits during threat responding, emotion processing, and empathic responding [16–23]. Prior meta-analyses in youth with conduct problems have grouped these processes into a general emotion processing category that includes all affective tasks [6, 10] or a “hot executive functioning” category that additionally includes cognitive tasks with affective components [9]. These meta-analyses have identified a range of differences with multiple meta-analyses identifying differences in the amygdala [9, 10], basal ganglia [6, 9, 10], and thalamus [6, 10]. Notably, however, no prior meta-analysis has investigated emotion-specific patterns of neural responding in youth with conduct problems, despite evidence that distinct affective domains like fear, anger, and pain recruit distinct networks of brain regions implicated in conduct problems [6, 9, 10, 24].

Prior meta-analyses have also investigated the specific impact of callous-unemotional traits on affective responding, with one meta-analysis reporting differences in the hypothalamus, thalamus, and ventromedial prefrontal cortex among youths with these traits [6]. Notably, these findings were found in youth with psychopathic traits generally, not callous-unemotional traits specifically, and across several emotion processing domains, rather than a specific type of emotion processing. A second meta-analysis conducting a region of interest (ROI) analysis found callous-unemotional traits were associated with reduced amygdala responding in both general affective and threat domains [25]. Our meta-analyses aimed to extend these findings by considering how conduct problems and callous-unemotional traits correspond to neural responses to affective and aversive imagery in general, as well as to four specific domains of socio-affective cues: negative images, fearful expressions, angry expressions, and empathic pain imagery [7]. Atypical responses to each of these socio-affective cues has been previously linked to the development of conduct problems.

We thus focused on studies that assessed responses to affective stimuli, including aversive images, fearful or angry facial expressions, and empathic pain images in youths with conduct problems ages 10–21. For each analysis, we compared all youths with conduct problems to controls, with additional analyses focusing on youths with callous-unemotional traits. We conducted both whole-brain analyses and ROI analyses of the left and right amygdala, the only ROI consistently reported across publications. We also conducted a follow-up meta-regression that included callous-unemotional traits to predict amygdala activity to fearful expressions. All hypotheses, included contrasts, sampling criteria, and analysis techniques were pre-registered at https://osf.io/wfegz.

Hypotheses included that youth with conduct problems would show (1) Reduced responding in prefrontal and limbic regions such as ventromedial prefrontal cortex, anterior cingulate cortex (ACC), and amygdala across task categories, (2) Reduced activity in the amygdala while viewing threatening/negative stimuli, (3) Reduced activity to empathic pain imagery within the pain matrix, such as the ACC anterior insula (AI), in youths with conduct problems, (4) Reduced activity in amygdala and ventromedial prefrontal cortex when viewing fearful expressions, with (4a) More severe reductions observed in the amygdala in youths with callous-unemotional traits.

## Methods

### Search procedure

Best-practices to identify fMRI studies for inclusion were followed [26]. A systematic literature search was conducted of whole-brain fMRI studies in youths (ages 0–21) with conduct problems with or without callous-unemotional traits published prior to February 2021. Original registration planned age cutoff at 18, we then increased the cutoff to 21 to capture a more complete sample of publications. The search was executed using PubMed, with search terms similar to those used in prior meta-analyses: “conduct disorder”, “oppositional defiant disorder”, “callous-unemotional”, “limited prosocial emotions”, “disruptive behavior”, “antisocial behavior”, “psychopathy”, and “psychopathic traits”, plus “functional magnetic resonance imaging”, and “fMRI” [6, 9, 10, 25, 27, 28]. The references of included articles were again examined for inclusion. This approach allowed for a thorough search of all current literature [6, 25, 27].

### Study selection

Articles were included if they (1) reported *x*/*y*/*z* coordinates using Talairach and Tournoux or Montreal Neurological Institute (MNI) templates [29, 30], (2) reported results based on whole-brain analyses, (3) included *z*-statistics, *t*-statistics, or uncorrected *p*-values, (4) reported statistics of peak activation in Talairach41 or MNI template space, (5) included more than 10 participants, (6) included participants ages 21 or younger, (6) were published in English, and (7) were published prior to February, 2021. Additionally, only studies with relevant contrasts were included (aversive/threating stimuli > neutral stimuli; fearful > neutral/happy/scrambled expressions, anger > neutral/happy/scrambled expressions, and self-pain > other-pain). Reward and punishment tasks were not included due to poor overlap across studies in available contrasts; additionally, too few studies included prediction error during reward processing for inclusion. Similar to other meta-analyses [6, 9, 10, 25, 27], we included only case–control studies in which healthy youth were compared to youth either diagnosed with a disruptive behavior disorder or described as having elevated conduct problems as assessed using a research instrument like the Child Behavior Checklist. This enabled planned analyses to test group differences. Studies were included in the callous-unemotional traits meta-regression analysis if they reported scores on a measure of callous-unemotional traits, including the Youth Personality Inventory Callous-Unemotional scale (YPI-CU) [31], the Inventory of Callous-Unemotional Traits (ICU) [32], or the Antisocial Process Screening Device Callous-Unemotional scale (ASPD-CU) [33].

**Fig. 1:**
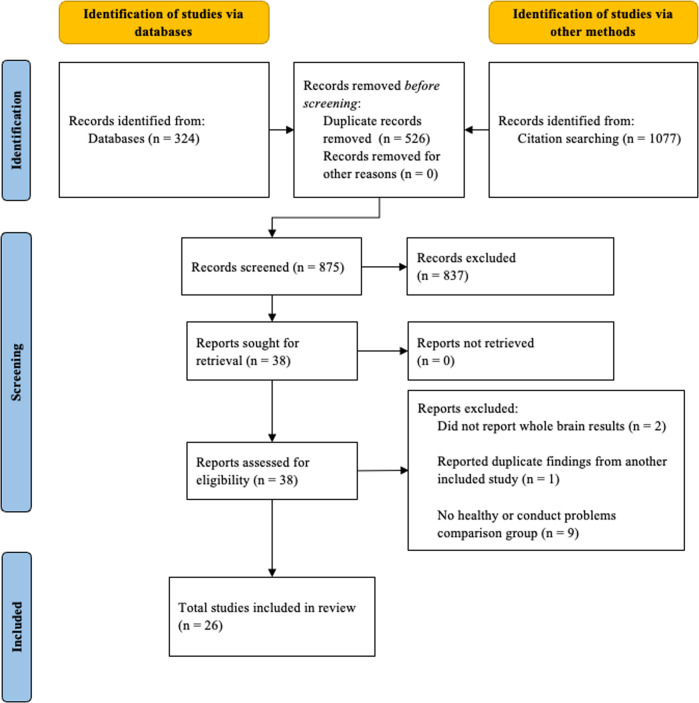
PRISMA 2020 flow diagram. After studies were identified from a database and citation search, duplicate records were removed. Full text records were screened and studies were excluded (837 studies) that summarized the literature and did not report new findings, we not within the age range, did not use task based fMRI to measure emotion processing, did not investigate conduct problems, or were case studies. Additional screening excluded studies that did not report whole brain results (2 studies), reported duplicate findings from an already included study (1 study), did not have a healthy or conduct problems comparison group (9 studies).

Prisma workflow guidelines were followed for article selection (Fig. 1). Articles were reviewed by two researchers (K.B., M.P.) for eligibility, and after the initial screening process 12 studies were excluded for the following reasons: 2 did not report any whole-brain contrasts [34, 35], 1 reported findings already reported in a separate included study [36], and 9 did not include both healthy controls and youths with conduct problems, precluding group comparisons [37–45].

### Meta-analysis pre-processing

We used seed-based d Mapping with Permutation of Subject Images (SDM-PSI version 6.21) to estimate effect-size maps, with lower and upper bounds, for all relevant contrasts collected based on peak coordinates and reported or calculated *t*-values [46]. We selected this method because it accounts for effect-sizes, null results, and includes sign information to counteract positive and negative differences between peaks. Family-wise error calculations also provide stricter significance testing. These stricter thresholds favor avoiding Type 1 errors and thus yield more accurate effect-size maps, but may result in more null findings than methods used in some previous meta-analyses. All whole-brain maps were calculated using gray matter masks. ROI analyses used the Automated Anatomical Labeling (AAL) Atlas with 2 mm [3] voxels. Voxel estimates were then imputed multiple times to reduce bias [47]. These images were combined into a single image for each study to be entered into the meta-analysis. Finally, corresponding voxels across all study images were combined into the final meta-analysis effect-size map. Results are reported separately that were statistically significant following FWER-correction threshold-free cluster enhancement (TFCE) (1000 permutations) (*p* < 0.05) and that were statistically significant at an uncorrected threshold of *p* < 0.005 [46].

To check for potential sampling error, we collected between-study heterogeneity statistics (*I*^*2*^), with a score of 25% or less reported as low. Funnel plots were also generated to visualize relative contributions of studies to meta-analytic findings, and publication bias statistics are reported for each significant peak.

The same method was used for all analyses to generate relevant contrasts. In the case of the main analysis, if multiple contrasts were available, as was the case for studies in which youths with both high and low callous-unemotional traits were included, or studies in which responses to both angry and fearful expressions were included, the contrast was selected that represented the largest sample and fearful contrasts were selected. Finally, we used an ROI approach to assess activation in the left and right amygdala. Other ROIs could not be assessed due to insufficient studies reporting common ROIs. Left and right amygdala ROIs were investigated separately using the AAL atlas [48].

To better assess the potential impact of callous-unemotional traits on amygdala responses to fearful facial expressions we conducted a meta-regression analysis. Due to inconsistencies in the measures used to assess callous-unemotional traits we calculated the percent of maximum possible (POMP) value for each study [49] a technique used in prior meta-analyses [25, 50].

## Results

### Characteristics of included studies

In total, 31 contrasts from 23 fMRI studies were included, which comprised 606 youths with disruptive behavior disorder diagnoses or described as having conduct problems (mean age = 14.59, mean age range = 11.9–17.45; mean% male = 73.24, mean IQ = 99.21) and 459 comparison youths (mean age = 14.59; mean age range = 11.30–17.80, mean% male = 69.41, mean IQ = 102.69) [19–23, 51–68]. Studies varied in the measures used to assess clinically relevant levels of conduct problems. Assessment information is reported in Table 1. Five studies including a total of 92 youth also reported separate contrasts specifically investigating responses to fearful facial expressions in youth with callous-unemotional traits relative to healthy controls (mean age = 14.02, mean age range = 11.9–15.5, mean% male = 84.72, mean IQ = 98.53).

**Table 1:** Summary of whole-brain fMRI studies of youths with conduct problems relative to healthy control youths included in functional meta-analyses by task domain.

Study	Year	Amygdala ROI	Contrast	Callous-unemotional					POMP	Assessment	Conduct problems				Assessment	Controls		%*M*	IQ
				*N*	Age	%*M*	IQ	Score			*N*	Age	%*M*	IQ		*N*	Age		
*Negative images*																			
Cohn	2013	X	CS+ > CS–	–	–	–	–	26.8	0.6	YPI-CU	25	17.5	72	88.95	DISC-IV	26	17.8	89	91.9
Fehlbaum	2018	X	Negative > neutral	–	–	–	–	11.2	0.25	YPI-CU	39	15.9	74.4	99.47	DSM-5	39	15.54	74.4	101.54
Herpertz	2008	X	Negative > neutral	–	–	–	–	–	–	–	22	14.7	100	96	CBCL	22	14.7	100	101
Hwang	2016	X	Violent words > non-violent words	–	–	–	–	48.1	0.67	ICU	35	14.8	62.8	96.19	K-SADS	28	13.88	53.5	101.18
Kalnin	2011	--	Violent words>	–	–	–	–	–	–	–	22	14.6	59	96.59	K-SADS	22	14.86	59	99.9
Raschle	2019	X	Look negative > look neutral	–	–	–	–	30.1	0.67	YPI-CU	30	16.3	0	99.9	K-SADS	29	16.74	0	106.24
Sterzer	2005	X	Negative > neutral	–	–	–	–	–	–	–	13	12.9	100	97.54	DSM-IV	14	12.71	100	117.36
Thornton	2017	X	Negative faces > Neutral faces	–	–	–	–	42.6	0.59	ICU	29	14.7	69	99.28	K-SADS	20	14.05	50	103.89
Whitec	2018	X	Threat > neutral	–	–	–	–	42.8	0.59	ICU	31	14.6	71	97.87	K-SADS	27	14.91	51.9	101.96
Whited	2014	--	Physical threat > appetitive	–	–	–	–	31.7	0.44	ICU	15	14.4	73.3	93.47	K-SADS	15	14.04	66.7	106.6
*Facial expressions*																			
Lozier	2014	X^a^	Fear > baseline	14	15.5	50	95.29	51.3	0.68	ICU	30	12.8	62.5	112.7	SDQ/CBCL	16	14.8	53	96.695
Fairchild	2019	--	Anger > neutral	–	–	–	–	–	–	–	20	17	0	99.65	K-SADS	20	17.62	0	104.6
Jones	2009	X	Fear > neutral	17	11.9	100	100.4	8.14	0.68	ASPD-CU	17	11.9	100	100.4	SDQ	13	11.3	100	100.54
Marsha	2008	--	Fear > neutral, Anger > neutral	12	14.2	50	104	^b^	^b^	YPI-CU	12	14.2	50	104	K-SADS	12	14.5	58.3	101
Passamonti	2010	--	Anger > neutral	–	–	–	–	^c^	^c^	YPI-CU	41	13	100	95.4	K-SADS	21	13	100	98.7
Sebastiana	2014	X	(Fear eyes > calm Eyes) > (fear face > calm face)	17	14	100	98.35	53.4	0.74	ICU	34	13.5	100	106.7	CASI-CD	17	14.27	100	100.62
Sebastianb	2021	X	Fear > calm, Anger > calm	17	14	100	97.41	51.2	0.71	ICU	34	14	100	101	CASI-CD/SDQ	18	14	100	102.83
Viding	2012	X	Fearful > calm	15	14.2	100	98.8	53.5	0.74	ICU	30	13.7	100	108.4	CASI-CD	15	14.46	100	101.27
Whitea	2012	--	Fear > neutral	17	15.5	76	90.88	27.3	0.68	ASPD-CU	17	15.5	76.4	90.88	K-SADS	19	15.22	47.3	110.47
Whiteb	2012	--	Fear > neutral, anger > neutral	15	15.7	80	96.67	29.1	0.73	ASPD-CU	15	15.7	80	96.67	K-SADS	17	14.5	52.9	101.19
*Empathic pain*																			
Decety	2009	--	Pain caused by other > Pain caused by accident	–	–	–	–	–	–	–	8	NA	NA	NA	DISC-CD	8	NA	NA	NA
Lockwood	2013	--	Pain > no pain	–	–	–	–	43	0.6	ICU	37	14.1	100	101.2	CASI-CD	18	13.68	100	102.83
Marshb	2013	--	Others pain > own pain	–	–	–	–	11.8	0.74	PCL-YV	14	15.4	57	100.5	K-SADS	21	14.3	71	106.9

*CU* Callous-unemotional, *CS* conditioned stimulus, *YPI* Youth Personality inventory, *ICU* Inventory of Callous-unemotional Traits, ASPD antisocial process screening device, *PCL-YV* Psychopathy Checklist Youth Version, *DISC-IV* Diagnostic Interview Schedule for Children Version Four, *DSM-5* The Diagnostic and Statistical Manual of Mental Disorders Fifth Edition; *CBCL* Child Behavior Checklist, K-SADS Kiddie Schedule for Affective Disorders and Schizophrenia, *SDQ* Strengths and Difficulties Questionnaire; *CASI-CD* Child and Adolescent Symptom Inventory Conduct Disorder Scale, DISC-CD Diagnostic Interview Schedule for Children Conduct Disorder.

^a^Only right amygdala values reported.

^b^Values not reported.

^c^Score reported was calculated using an unknown transformation.

### Group differences across all studies

The whole-brain meta-analysis included 26 contrasts, one selected from each included study, and spanned all task types. No regions survived whole-brain FWER-correction (*p* < 0.05) and only a significant uncorrected finding in the left cerebellum was observed (Table 2).

**Table 2:** Results of whole-brain and ROI fMRI meta-analyses of youths with conduct problems relative to healthy control youths by task domain and presence of callous-unemotional traits.

Meta-analysis	*L*/*R*	MNI coordinate	SDM-Z	Voxels	*P* (uncorrected)	*P* (corrected)
*All tasks*						
*Conduct problems * *>* * healthy controls*						
Cerebellum, hemispheric lobule IV/V	*L*	−4, −50, 0	2.65	30	0.004	–
Amygdala ROI						
*Healthy controls* * >* * conduct problems*						
Amygdala	*R*	28, −2, −18	−4.425	198	<0.001	–
*Negative images*						
*Amygdala ROI*						
*Healthy controls* > *conduct problems*						
Amygdala	*R*	28, −2, −18	−3.467	50	<0.001	–
*Fearful facial expressions*						
*Amygdala ROI*						
*Healthy controls* > *conduct problems*						
Amygdala	*R*	30, 0, −26	−2.862	6	0.002	–
*Fearful facial expressions—callous unemotional traits*						
*Healthy controls* > *conduct problems*						
Middle temporal gyrus	*L*	−54, −8, −20	−2.728	17	0.003	–
Superior parietal gyrus	*L*	−24, −68, 46	−3.371	46	<0.001	–
Fusiform gyrus	*L*	−40, −74, −16	−2.994	18	0.001	–
*Angry facial expressions*						
Supplementary motor area	*L*	−2, 22, 44	−3.331	122	<0.001	–
*Empathic pain*						
*Conduct problems* > *healthy controls*						
Hippocampus	*L*	−18, −20, −16	4.279	27	<0.001	–
Cerebellum, vermic lobule IV/V		−2, −48, −4	3.533	21	<0.001	–
*Healthy controls* > *conduct problems*						
Middle frontal gyrus	*R*	22, 48, 24	−3.534	28	<0.001	–
Inferior frontal gyrus, triangular part	*R*	54, 38, 0	−3.695	74	<0.001	–
Inferior frontal gyrus	*L*	−36, 34, 20	−3.608	38	<0.001	–
Supplementary motor area	*L*	−10, 12, 52	−3.486	17	<0.001	–
Temporal pole, middle temporal gyrus	*R*	44, 8, −36	−3.682	115	<0.001	0.021
Thalamus	*L*	−4, −14, 18	−3.356	8	<0.001	–
Superior temporal gyrus	*L*	−42, −18, −6	−3.622	11	<0.001	–
Cerebellum, vermic lobule IV/V		0, −48, −20	−3.818	89	<0.001	–
Hemispheric lobule VI		30, −56, −30	−3.577	66	<0.001	0.006
Precuneus	*L*	−4, −62, 26	−3.471	40	<0.001	–
Cerebellum, Crus I	*R*	14, −72, −32	−3.991	63	<0.001	0.003

Our ROI analysis included 25 contrasts (one study did not report left amygdala results) with 5 studies reporting responses to fearful facial expressions and 8 reporting responses to negative images (Table 1). Results showed reduced activation in right amygdala in youths with conduct problems relative to controls (Table 2). No results were observed in left amygdala. Follow-up meta-regression analysis including POMP callous-unemotional scores from each study revealed, among youths with conduct problems, no relationship between right amygdala activation across tasks and callous-unemotional traits.

### Group differences for negative images

The whole-brain meta-analysis of studies reporting responses to negative affective images included 10 contrasts, one selected from each included study (Table 1). No findings that survived FWER-correction or significant uncorrected findings at *p* = 0.005 were observed.

**Fig. 2:**
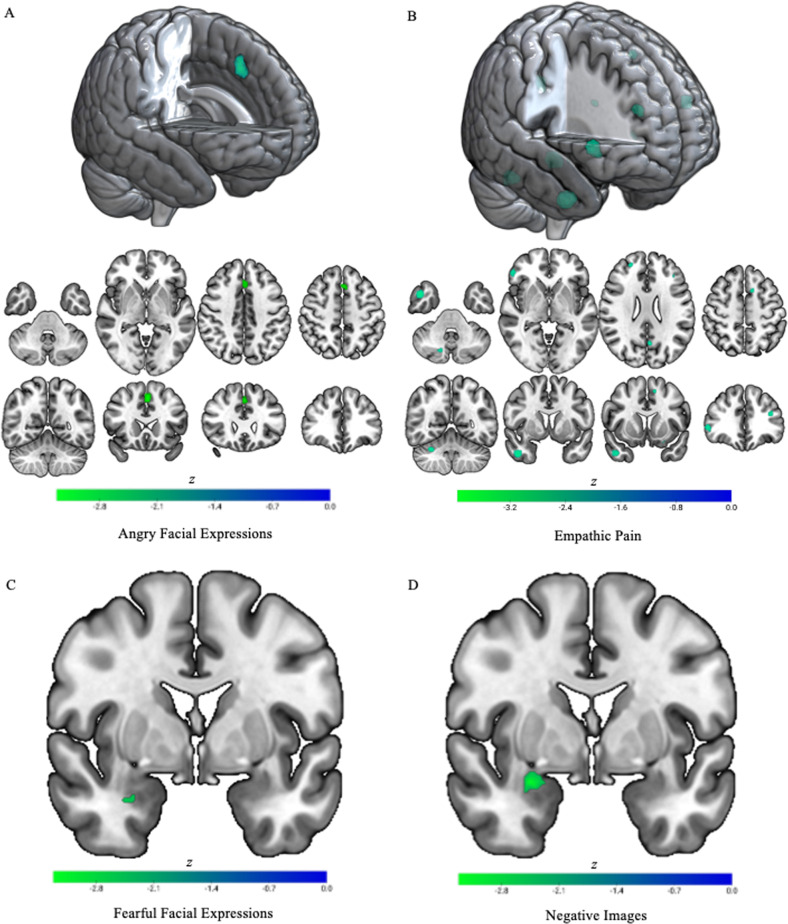
Regions reduced in youths with conduct problems relative to healthy control youths in whole-brain and ROI fMRI meta-analyses by task domain. **A** Whole-brain results on angry facial expressions. **B** Whole-brain findings on empathic pain responses. **C** Results from the amygdala region of interest analysis on fearful facial expressions. **D** Results from the amygdala region of interest analysis on negative images. The right side of the image corresponds to the left side of the brain.

The ROI analysis included 16 contrasts, with 2 (left and right amygdala) selected from each included study (Table 1). Again, results showed reduced activation in right amygdala in youths with conduct problems relative to controls (Table 2 and Fig. 2). No results were observed in left amygdala. Follow-up meta-regression analysis including POMP callous-unemotional scores from each study revealed that among youths with conduct problems, no relationship was observed between right amygdala activation to negative images and callous-unemotional traits.

### Group differences for fearful facial expressions

The whole-brain meta-analysis of studies reporting responses to fearful facial expressions included 8 contrasts, one selected from each included study and revealed no findings that survived FWER-correction or significant uncorrected findings at *p* = 0.005.

The ROI analysis included 9 contrasts with one study not reporting left amygdala findings (Table 1). Again, results showed reduced activation in right amygdala in youths with conduct problems (Table 2 and Fig. 2) and no results were observed in left amygdala. Follow-up meta-regression analysis including POMP callous-unemotional scores from each study revealed no relationship between right amygdala activation when viewing fearful expressions and callous-unemotional traits among youth with conduct problems.

A second whole-brain meta-analysis was also conducted examining only responses among youths with conduct problems and high levels of callous-unemotional traits to fearful facial expressions, which included 8 contrasts, one selected from each included study (Table 1). No results survived FWER-correction. Regions in which youths with conduct problems showed less activation at an uncorrected threshold (*p* < 0.005) included a cluster spanning left superior and inferior parietal gyri, left fusiform gyrus, and left middle temporal gyrus (Table 2).

### Group differences for angry facial expressions

The whole-brain meta-analysis of responses to angry facial expressions images included five contrasts, one selected from each included study. No results survived FWER-correction. Uncorrected group differences in activation were observed in a cluster spanning left supplementary motor area and superior frontal gyrus (Table 2 and Fig. 2). ROI analyses were not conducted due to a lack of sufficient available studies (*n* = 1).

### Group differences for empathic pain images

The whole-brain meta-analysis of responses to empathic pain images included 3 contrasts (Table 2). Three regions survived FWER-correction, including a region that spanned right temporal pole and included areas of middle and inferior temporal gyrus in which youths with conduct problems showed reduced activation (Fig. 2). Regions in which uncorrected group differences emerged included one region, left hippocampus, in which youths with conduct problems showed increased activation relative to controls, and several regions in which youths with conduct problems exhibited reduced activation, including two regions of the pain matrix: left SMA and thalamus and several regions of frontal and temporal cortex (Table 2 and Fig. 2). ROI analyses were not conducted due to too few available studies (*n* = 0).

### Reliability analyses

Analysis of between-study heterogeneity across all studies revealed whole-brain analysis peaks and ROI peaks showed low between-study heterogeneity (whole-brain: *I*^2^ = 0.2–14.8%; ROI: *I*^2^ = 0.83%) confirming results were not likely driven by sampling error. Additionally, we found low likelihood of sampling error in each socio-affective specific meta-analysis including negative images (*I*^2^ = 1.3%), fearful facial expressions (ROI: *I*^2^ = 3.8%; callous-unemotional traits: *I*^2^ = 1.9%–3.8%), angry facial expressions (*I*^2^ = 4.2–1.8%), and empathic pain images (*I*^2^ = 0–4.5%).

### Publication bias

There was no significant evidence of publication bias across all studies (whole-brain: *p* = 0.712–0.816; ROI: *p* = 0.723) or within socio-affective categories (negative: *p* = 0.971; fearful facial expressions ROI: *p* = 0.538, fearful facial expressions callous-unemotional traits: *p* = 0.793–0.985; angry facial expressions: *p* = 0.888–0.946). An insufficient number of studies existed to test for bias in the empathic pain meta-analysis. An investigation of the funnel plots across all meta-analyses did not suggest any one study contributed more heavily to significant peaks.

## Discussion

This meta-analysis included both whole-brain and amygdala ROI results from over 1000 youth (23 studies) and, to our knowledge, is the first meta-analysis to investigate emotion-specific differences in youths with conduct problems. Results consistently indicated that youths with conduct problems show reduced responding in right amygdala when viewing a range of negative affective images, including threatening images and fearful facial expressions. Results also make clear that responses to negative affective images are not interchangeable, with distinct patterns of atypical activation observed in response to the various sub-categories of images. When viewing angry expressions, youths with conduct problems exhibited reduced activation in left SMA; by contrast, when they observed fearful expressions, no differences were seen in this region. We also observed reduced activation in the left superior parietal gyrus, left fusiform gyrus, and left middle temporal gyrus in high callous-unemotional youths specifically when they viewed fearful faces. Finally, we found reduced activation in a subset of regions within the canonical pain matrix, including the thalamus and SMA (although notably not the anterior insula or dorsal anterior cingulate cortex, as we had hypothesized), as well as temporal pole when youth with conduct problems viewed images of others in pain.

In general, our most consistent finding was that youths with conduct problems exhibited reduced activation in right amygdala, specifically in response to negative images and fearful facial expressions. This finding is consistent with theories regarding etiologies of conduct problems [7] and replicates several other meta-analytic findings in both youth [9, 10, 25] and adults [25, 28]. Reduced amygdala response to negative images may underpin youth with conduct problems’ difficulty responding to fear-relevant stimuli, including difficulties coordinating physiological and cognitive responses to threats, and difficulties in interpreting social fear cues [17, 69–74]. Our findings are consistent with the interpretation that reduced responding in the amygdala to a range of fear-relevant cues may underlie insensitivity to threat in children with conduct problems, which increases their likelihood of engaging in physically or socially risky behaviors that may result in punishment [72]. Reduced amygdala responsiveness to fearful expressions may also impair the ability of youths with conduct problems to empathize with and correctly interpret others’ fear [70, 71, 75], and to avoid behaviors likely to cause fear in others [76]. According to simulation theories of empathy, observers recruit the same networks when observing others’ emotions that they recruit when personally experiencing those emotions [77]. Thus, just as the amygdala plays a pivotal role in coordinating personally experienced fear [78], it may play a similarly pivotal role in empathizing with others’ fear.

It is noteworthy that, inconsistent with our hypothesis and prior meta-analytic findings [25], reduced amygdala responding was found across youths with conduct problems, rather than being limited to youths with higher callous-unemotional traits. This finding could indicate that youths with conduct problems, even those without high callous-unemotional traits, generally show reduced amygdala responsiveness relative to healthy controls [23]. This may in part reflect the fact that callous-unemotional traits and externalizing behavior are highly correlated [79, 80]. In addition, several included studies compared control youths only to youths with conduct problems and callous-unemotional or psychopathic traits [19, 62, 66, 67]. In these studies, even youths with *lower* levels of callous-unemotional traits nonetheless had relatively high baseline callous-unemotional traits. Thus, it is possible that included studies were not sufficiently powered, due either to sample size or range restriction, to identify patterns of neural variation that correspond to variation in callous-unemotional traits among children with conduct problems. Relatedly, our meta-regression found no relationship between callous-unemotional traits and fearful facial expressions. However, POMP scores ranged from .68-.74, meaning no study reported findings where youth averaged callous-unemotional traits scores in the top 25 percent of their respective scale. To better assess the true impact of callous-unemotional traits on fear processing, future research should be clear about the threshold that qualifies youth for “high” levels of callous-unemotional traits.

We were able to conduct a separate whole-brain analysis for youth with high levels of callous-unemotional traits that revealed additional differences when these youths view fearful facial expressions, including reduced activity in left superior parietal gyrus, left fusiform, and left middle temporal gyrus. In contrast to prior meta-analytic results, we did not find differences in the middle frontal gyrus [6]. These differences could be due to the specificity of our analysis which only included youth with callous-unemotional traits and not psychopathic traits more broadly; additionally, we tested differences in responding to fearful expressions rather than across all emotion processing tasks. Consistent with previous meta-analyses, we found differences in left fusiform activation that have also been reported in other meta-analysis that include individuals with conduct problems [9] and a population expanded into adulthood [25]. Although right fusiform is often recruited during face processing, a meta-analysis of facial affect responding also found left fusiform activation when healthy adults respond to negative faces [81]. Our findings of reduced activation in this region in youths with high levels of callous-unemotional traits may indicate differences in attention or face processing coupled with amygdala hypoactivation [71, 82, 83]. Again, callous-unemotional scores were limited to a relatively small range (0.68–0.74) and therefore, future research should investigate these regions in groups of youth scoring in the top 25 percent of callous-unemotional trait severity.

Distinct patterns were observed across samples in response to angry stimuli, confirming that it may not be appropriate to collapse these expressions together into a common “threat expression” category [84]. In responses to angry expressions, youth with conduct problems exhibited reduced activity in middle temporal gyrus and SMA. Although we did not have specific hypotheses regarding responses to angry facial expressions, differences in SMA activation to threating stimuli have also been reported in a previous meta-analysis that included both youth and adults with conduct and antisocial behavior problems [25]. This may may be in part because SMA is adjacent to and is often co-activated with, anterior mid-cingulate cortex, as it is in the current meta-analysis: The SMA cluster we identified extends into the anterior mid-cingulate cortex. These regions activated in conjunction with one another are important for regulating approach and avoidance behaviors, as well as error detection and response selection [85, 86]. Angry expressions are believed to function in social interactions to signal social dominance and motivate others to change their behavior [69, 72]. Reduced responding in this region in youth with conduct problems to others’ anger may be underlie their reduced tendency to adjust their behavior in response to these social signals, an interpretation consistent with increased social dominance and aggression seen in youth with conduct problems [69, 72].

Consistent with our hypotheses, we found youths with conduct problems exhibit reduced activity to empathic pain imagery in two canonical pain regions: SMA and thalamus. SMA and thalamus are regions consistently recruited during acute pain [16, 87], as well as when observing others in pain [88, 89]. These regions have also been found in other meta-analyses investigating antisocial behavior and conduct problems [10, 25, 27]. Our results are therefore generally consistent with impairments in empathic pain responding in youths with conduct problems, which may contribute to their increased aggression. However, given the limited number of studies included in the meta-analysis, these results should be interpreted with caution. Neurodevelopmental changes in this circuit may contribute to aggression and social learning impairments in youth with conduct problems [72], a possibility that should be considered in future research on this topic.

Interestingly, the empathic pain meta-analysis produced the most overlap in brain regions when compared to other emotion-general meta-analyses. Other studies report overlapping findings in the superior [10] and inferior frontal gyrus [25, 27], hippocampus [25, 27], temporal pole [10], thalamus [10], and precuneus [27]. The large amount of overlap between prior emotion-general meta-analyses and empathic pain highlights the conducting meta-analyses with emotion-specific sub-analyses. The greater consistency in the methods of included studies may explain why the most robust findings were also observed when examining group differences during empathic pain tasks while other findings did not survive FWER-correction. Prior studies utilizing the same analysis technique as was used here (SDM) either do not report FWER-corrected results [6] or also report few regions surviving error correction [25]. It is possible this technique, SDM-PSI, is more sensitive to null findings and effect-sizes reported in individual studies, which may highlight the need for larger neuroimaging studies in the future with greater sensitivity. Also highlighting the need for larger studies, group differences in several hypothesized regions, including the insula and prefrontal cortex, were not found in the meta-analysis, despite frequent reports of functional differences in these regions between youth with and without conduct problems [7]. Larger fMRI studies with more power will allow future emotion-specific meta-analyses to more precisely assess the robustness of these meta-analytic findings and may identify other functional differences in, for example, core regions of the pain matrix like anterior insula and dorsal anterior cingulate cortex.

These findings should be interpreted in light of certain limitations. First, the sample was drawn from a heterogenous set of research studies in terms of sampling and empirical design. Other variables that may distinguish subgroups of youths with conduct problems such as symptom onset, or exposure to trauma—were rarely distinguished, preventing consideration of the potential role of these variables [90–93]. We were also unable to consider the role of disorders that often co-occur with conduct problems (including ADHD, anxiety, and depression). Finally, we were not able to control for variations among the tasks we included, such as whether they featured implicit versus explicit processing or passive viewing of stimuli; these specific task features may alter recruitment of amygdala and other regions [81].

Our findings highlight differences in processing socio-affective cues including fearful and angry expressions and empathic pain that may underpin a lack of empathic responding, poor regulation of approach and avoidance, and heightened risk for aggression [17, 20, 86, 94]. Future research should employ larger sample sizes for greater power and recruit youth with more variable levels of callous-unemotional traits to investigate the amygdala, SMA and mid-cingulate, as well as the left fusiform as potential targets for interventions aimed at reducing aggression and antisocial behavior.
